# Impact of early versus late enteral nutrition on cell mediated immunity and its relationship with glucagon like peptide-1 in intensive care unit patients: a prospective study

**DOI:** 10.1186/cc12795

**Published:** 2013-06-20

**Authors:** Okan Bakiner, Emre Bozkirli, Semih Giray, Zulfikar Arlier, Ilknur Kozanoglu, Nurzen Sezgin, Cagla Sariturk, Eda Ertorer

**Affiliations:** 1 Baskent University, Faculty of Medicine, Department of Endocrinology and Metabolism Diseases, Fatih Sultan Mh., 06990 Ankara/Ankara Province, Turkey; 2 Baskent University, Faculty of Medicine, Department of Neurology, Fatih Sultan Mh., 06990 Ankara/Ankara Province, Turkey; 3 Baskent University, Faculty of Medicine, Department of Physiology, Fatih Sultan Mh., 06990 Ankara/Ankara Province, Turkey; 4 Baskent University, Faculty of Medicine, Department of Biochemistry, Fatih Sultan Mh., 06990 Ankara/Ankara Province, Turkey; 5 Baskent University, Faculty of Medicine, Department of Biostatistics, Fatih Sultan Mh., 06990 Ankara/Ankara Province, Turkey

## Abstract

**Introduction:**

Glucagon-like peptide-1 (GLP-1) originates from the gastrointestinal system in response to the presence of nutrition in the intestinal lumen and potentiates postprandial insulin secretion. Also, it acts as an immune-modulator which has influences on cell-mediated immunity.

The aim of this study was to determine the impact of early enteral nutrition versus late enteral nutrition on plasma GLP-1 levels and the relationship between GLP-1 changes and cell-mediated immunity.

**Materials and methods:**

The study was designed as a prospective, single-blinded study and carried out in the neurology intensive care unit (ICU) of a university hospital. Twenty-four naive patients with acute thromboembolic cerebrovascular events, with National Institute of Health (NIH) stroke scores between 12 and 16, were included. Any condition interfering with GLP-1 and immunity was regarded as exclusion criterion. Two patients died, and two dropped out of the study due to complicating conditions.

Patients were randomly subjected to early enteral feeding within the first 24 hours (Group 1), or late enteral feeding, beginning 48 hours after admission (Group 2) via a nasogastric tube. Calculated daily energy requirement was supplemented with parenteral nutrition, starting on the first study day for both groups. Blood samples were obtained before, and at 5, 15, 30, 60 and 120 minutes after the first enteral feeding for GLP-1 assays; this procedure was repeated on the third day. Before and 24 hours after the first enteral feeding, samples were also taken for immunological analysis. Clinical observations were recorded.

Pre- and post-feeding plasma GLP-1 changes between the two groups and within groups were evaluated. Lymphocyte subgroup changes before and 24 hours after the first enteral feeding in relation to GLP-1 changes were sought as well.

**Results:**

Group 1 and Group 2 exhibited similar GLP-1 levels in the pre-feeding and post-feeding periods for both the first time and the third day of enteral feeding. Also, no significant change in pre-/post-feeding GLP-1 levels was observed within groups. T-helper and T-regulatory cells increased, T-cytotoxic cells decreased significantly in Group 1 (*P *= 0.02; *P *= 0.036; *P *= 0.0019), but remained the same in Group 2 after enteral feeding. Positive but statistically insignificant clinical effects in terms of predisposition to infections (10% vs 40%) and median time of ICU stay (10 vs 15 days) were observed in Group 1.

**Conclusions:**

Depending on our findings, we propose that early enteral feeding may cause amelioration in cell-mediated immunity via factors other than GLP-1 in ICU patients with acute thromboembolic stroke. However, the possible deleterious effects of parenteral nutrition cannot be ruled out.

## Introduction

A hyper-catabolic state and resulting protein energy malnutrition are closely associated with mortality among patients in intensive care units (ICU) [1]. Introduction of early enteral nutrition within the first 24 to 48 hours after admission has been demonstrated to decrease septic complications, ameliorate the course of primary disease and shorten the stay in the ICU when compared with parenteral nutritional support [2-6]. These positive findings were attributed to the prevention of worsening in intestinal permeability, interruption of the catabolic process and restoration of immune response [7].

Incretin hormones originate from the gastrointestinal system in response to the presence of nutrition in the intestinal lumen and potentiate postprandial insulin secretion. Glucagon-like peptide-1 (GLP-1) is the best known incretin hormone and is secreted principally from the L-cells of the distal ileum. The main stimulant of GLP-1 secretion is the presence of food awaiting absorption in the intestinal lumen [8]. In a recent study, GLP-1 has been demonstrated to act as an immune-modulator and influence cell-mediated immunity [9]. Its receptors have been shown on T-receptors in animal models, and their stimulation with supraphysiological concentrations of GLP-1 has been demonstrated to regulate the proliferation of lymphocytes and peripheral T regulatory (TREG) cells [10,11]. The TREG cells originate from the thymus and play a pivotal role in the prevention of autoimmune diseases by establishing immune tolerance. They control immunity-mediated injury of the host by limiting inflammation and tissue damage [12]. They have also been demonstrated to act as an immune-modulator following acute stroke, and to limit the extent of the damaged tissue area by controlling cellular inflammation [13].

To our knowledge, there has been no study inquiring about the relationship between early enteral nutrition and changes in plasma GLP-1 levels, and also the resulting clinical implications among ICU cases. In this clinical trial, our aim was to determine the impact of early enteral nutrition within the first 24 hours of admission, and late enteral nutrition; beginning 48 hours after admission, on plasma GLP-1 levels of acutely ill ICU patients with thromboembolic stroke. The possible relationship between the changes in GLP-1 and cell-mediated immunity was sought, as well.

## Materials and methods

### Patient selection

Patients between 40 and 70 years old admitted to our neurology ICU with the diagnosis of an acute thromboembolic cerebrovascular event were included in the study. Admission within the first 24 hours of the onset of symptoms with any treatment and exhibiting National Institute of Health (NIH) stroke scores [14] ranging between 12 and 16 were strictly sought for each individual at inclusion.

Accompanying pregnancy, diabetes, renal or hepatic failure, malignancy, chronic inflammatory bowel disease, previous gastrointestinal resection for any cause, and use of any medication that might interfere with immune system were regarded as exclusion criteria. Concomitant fluid and electrolyte imbalance, gastroparesis of any etiology were also considered as ineligibility.

This study was approved by the Ethics Committee of Baskent University (Project no: KA11/63) and was supported by the Baskent University Research Fund.

Informed consent was obtained from first degree relatives of the participants.

Criteria for being dropped before completion of the study period were determined as:

- Death from any cause,

- Onset of multi-organ failure, renal or hepatic failure, adult respiratory distress syndrome (ARDS),

- Co-presentation of any two systemic inflammatory response syndrome (SIRS) symptoms [15],

- Detection of microorganisms in blood cultures in addition to SIRS,

- Obligatory cessation of nasogastric intubation due to aspiration pneumonia or any other cause,

- New-onset medical conditions indicating the use of any medication that was not included in the standard treatment protocol of stroke, such as glucocorticoids that might interfere with the immune system.

### Study protocol

**Figure 1:**
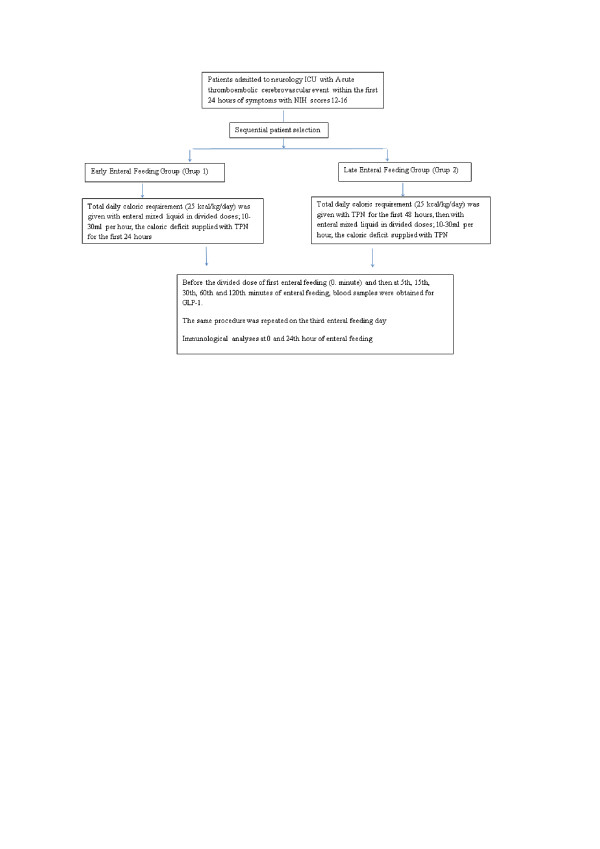
**A scheme demonstrating the study protocol**.

The study was designed in a prospective and single-blinded fashion (Figure 1). Patient allocation was performed sequentially and eligible patients were separated into two groups:

Group 1: Those who were subjected to enteral feeding within the first 24 hours of admission (early enteral feeding group)

Group 2: Those who were fed with total parenteral nutrition within the first 48 hours of admission, then were subjected to enteral feeding (late enteral feeding group)

The European Society of Parenteral and Enteral Nutrition (ESPEN) guideline for enteral and parenteral nutrition in intensive care units, which had been already in use in our ICU, was used [16,17]. Accordingly, daily caloric requirement was determined as 25 kcal/kg/day for each participant.

Enteral nutrition was supplied via a nasogastric tube (NGT) which was inserted by an ICU physician.

The early enteral feeding group (Group 1) was given the commercially available enteral nutrition liquid (JEVITY^® ^Abbott) within the first 24 hours via nasogastric intubation. Every 100 ml of this liquid contained 105 kcal energy with a combination of 14.05 g carbohydrates, 3.07 g fat and 4 g protein. Keeping with the ICU algorithm, enteral nutrition was started slowly with divided doses [16,18]; 10 ml/h (10.5 kcal/h) for the first six hours on the first day of enteral feeding, then 20 ml/h (21 kcal/h) for the following 12 hours and 30 ml/h (31.5 kcal/h) thereafter. The deficit in daily energy requirement was supported with supplemental parenteral nutrition (PN). The late enteral feeding group (Group 2) was given the daily recommended caloric requirement (25 kcal/kg/day) parenterally with the parenteral nutritional solution for the first 48 hours. Following this period, enteral nutrition was started using the same protocol mentioned for Group 1. In order to avoid caloric deprivation and complete the daily recommended caloric requirement, supplemental parenteral nutrition was given to both groups during enteral nutrition. To enable gastric tolerance, the amount of calories gained from enteral nutrition was 60% of the calculated calories within the first 72 hours and the deficit was supplied with PN as mentioned above. Details of daily nutritional support are given in Table 1.

**Table 1:** Details of daily energy supply of Group 1 and Group 2 regarding to EN and PN contribution (mean ± SD)

	GROUP 1		GROUP 2	
**#Study day**	**Enteral N (kcal)**	**Parenteral N (kcal)**	**Enteral N (kcal)**	**Parenteral N (kcal)**

**#1**	525 21	1,520 ± 35	-	1,950 ± 75
**#2**	790 ± 28	1,220 ± 24	-	1,950 ± 75
**#3**	1,025 ± 24	985 ± 50	515 ± 24	1,435 ± 21
**#4**	1,245 ± 50	815 ± 24	755 ± 21	1,185 ± 50
**#5**	1,560 ± 74	505 ± 78	1,015 ± 42	960 ± 24
**#6**	1,805 ± 63	255 ± 51	1,200 ± 54	755 ± 50
**#7**	2,045 ± 102	-	1,510 ± 74	445 ± 67

Before the first divided dose of the first enteral feeding (0. minute) and then at 5, 15, 30, 60 and 120 minutes after feeding, venous blood samples were obtained, centrifuged and stored at -40°C for GLP-1 analysis in both the early and late feeding groups. The same procedures were repeated on the third day after the first enteral feeding. Capillary glucose measurements were performed with a glucometric method before and after two hours of enteral feeding for all cases.

Additional blood samples in tubes containing EDTA were obtained from both of the groups, before the first enteral feeding and 24 hours later for immunological analysis. Leukocyte, lymphocyte count and CD4+/CD25high/foxP3+ (TREG cells), CD3 (total T lymphocytes), CD4 (T-helper cells) and CD8 (cytotoxic T cells) cell percentages were calculated by flow-cytometry device.

Daily NIH stroke scores of all patients were recorded [14]. The number of patients who required mechanical ventilation, time spent on mechanical ventilation and in ICU, and the survival rates were recorded. Patients who exhibited features of infection and required antibiotics were documented.

### Analyses

Glucagon-like peptide-1 assays were performed using a GLP-1 (active) enzyme-linked immunoassay kit (cat # EGLP-35K, Linco Research, Inc., St. Charles, MO, USA). The lowest reported detection limit was 2 pmol/l; reported within-assay coefficient of variation (CV) was 8% at low and high concentrations (range 4 to 76 pmol/l), and between-assay CV was 12% at 4 to 8 pmol/l and 7% at 28 to 76 pmol/l [19]. For GLP-1 ELISA assay blood samples were collected in ice-cooled Vacutainer^® ^EDTA-plasma tubes. DPP-IV inhibitor (Linco Research, Inc., Cat # DPP4, 10 μl DPP-IV inhibitor per milliliter of blood) was added immediately (<30 seconds) after collection to prevent enzymatic degradation of GLP-1. Tubes were inverted to mix and placed in an ice bath, and within 30 minutes were centrifuged at 1,000 x g for 10 minutes in a refrigerated centrifuge. Then plasma specimens were stored at -70°C until analysis.

Capillary glucose measurements were performed with the Medisense Precision QID (*r *= 0.979 with "YSI23AM Glucose Analyzer").

### Flow cytometric gating and analyses

**Figure 2:**
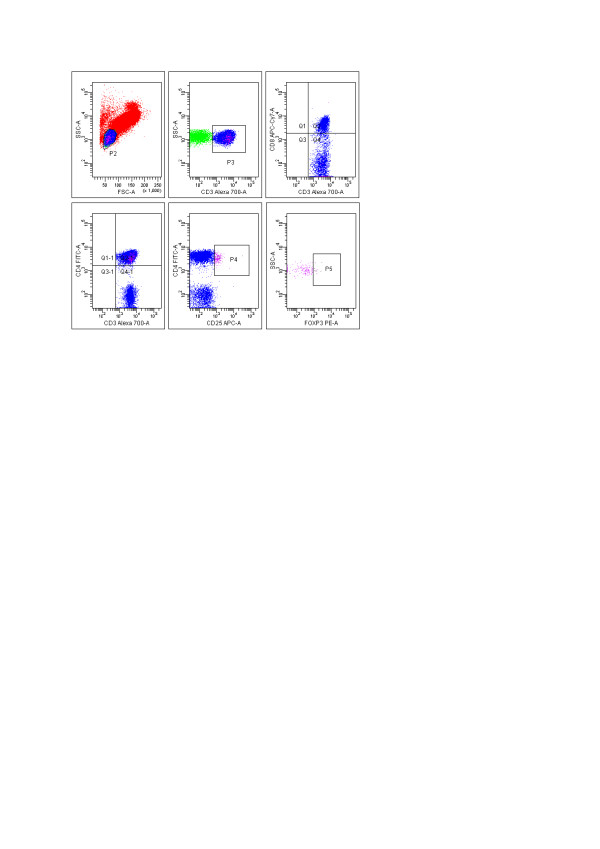
**Characterization of T lymphocyte, T-helper, T-cytotoxic and foxP3 expressions**. Lymphocytes had been selected from side/forward scatter and CD3, CD3CD4, CD3CD8 cells detected by using sequential gating. CD25+ foxP3+ TREG cells were shown within the CD4+ T cell population.

Data were analysed with FACS DIVA software (San Jose, CA, USA). All reagents and solutions were supplied from BD BioScience Company (San Jose, CA, USA). Gating analyses were performed in a blinded fashion. Cells were gated for the analyses of lymphocytes by side/forward scatter and gating for T cells was based on CD3 expression. Thereafter, the CD3 positive and CD4 positive cells were grouped in T helper (Th) and CD3 positive and CD8 positive cells (Tc) in T cytotoxic population. Gates for expression of CD25 in the CD4 population (CD4+CD25+) were selected and FoxP3 expressions were detected in these cells (CD4+CD25+Fox P3+) (Figure 2).

### Statistical analyses

A power calculation was conducted to determine the number of samples for cases required to perform this study in order to achieve 80% power for the GLP difference in an average of 0.2 at 5% significance level. This was achieved by using the EPI Info software package version 3.5.1. The minimum required number of patients was determined as 20. Statistical analyses were performed using the statistical package SPSS v 17.0. For each continuous variable, normality was checked by Kolmogorov Smirnov and Shapiro-Wilk tests. Comparisons between groups were applied using one-way Student's *t-*tests for normally distributed data and Mann Whitney test was used for the data not normally distributed. The categorical variables between the groups were analysed by using the Chi square test. The GLP-1 and other pre-post measured data were processed using the Repeated Measure Analyses. Receiver operating characteristic curves (ROC curves) were constructed and the areas under the curve (AUC) were calculated. Values of *P *< 0.05 were considered as statistically significant.

## Results

Twenty-four eligible cases out of 67 admissions were recruited between December 2011 and May 2012. During follow-up, two patients died before the end of the study period (one patient from each group). One patient developed acute renal failure (in Group 2) and one had aspiration pneumonia in the ICU (in Group 1); they were taken out of the study.

From a total of 20 patients, 10 in each group completed the study. The groups were compared according to age, gender, initial blood glucose level, NIH stroke score on admission, baseline GLP-1 level, total leucocyte and lymphocyte counts, CD3 (T lymphocyte), CD3CD4 (T helper cell), CD3CD8 (T cytotoxic lymphocyte) and TREG cell amounts as exact numbers on admission. No statistically significant difference was found (Table 2).

**Table 2:** Baseline characteristics of groups and comparison of study parameters

	Group 1 (*n *= 10)	Group 2 (*n *= 10)	*P*
**Male (M)/Female (F)**	7 M/3 F	5 M/5 F	--
**Age (years)**	67.7 ± 10.6	65.9 ± 10.8	0.712
**NIH stroke score**	14.5 ± 2.3	14.8 ± 1.1	0.718
**Capillary glucose (mg/dl)**	135.9 ± 16.6	141.8 ± 25.4	0.545
**GLP-1 (pmol/l)**	7.10 ± 3.52	8.57 ± 3.91	0.389
**Lymphocyte (n/mm3)**	681.95 (321.9 to 3745.4)	688.2 (490.5 to 2,014)	0.479
**Leukocyte (n/mm3)**	11.8 (8.3 to 20.2)	10.6 (4.5 to 18.5)	0.894
**CD3 cells (cell/μl)**	414.15 (167.39 to 2,857.74)	476.8 (296.3 to 1,208.4)	0.876
**CD3CD4cells (cell/μl)**	229.17 (96.9 to 1,583.1)	269.27 (136.4 to 821.7)	0.674

The daily median energy requirement in the Early Enteral Feeding Group was determined as 2,050 (minimum: 1,700 to maximum: 2,225) kcal/day. On the first day of feeding, 525 ± 21 kcal of it was supplied from enteral liquid and 1,520 ± 35 kcal from PN nutrition liquid. In other words, 25% of the calculated energy requirement was obtained from enteral liquid. On the other hand, the daily median energy requirement in the Late Enteral Feeding Group was determined as 1,950 (minimum 1,650 to maximum 2,375) kcal/day. The energy needs of this group were supported only by the total parenteral nutrition for the first 48 hours. Total energy supplied on the first enteral feeding day was statistically indifferent between the two groups (*P *= 0.37).

### Comparison of changes in GLP-1 levels between the two groups and within groups

Documentation of median (minimum to maximum) GLP-1 measurements with regard to time points of enteral feeding both in Group 1 and Group 2 are given in Table 3. The early enteral feeding group (Group 1) and late enteral feeding group (Group 2) exhibited similar plasma GLP-1 levels before the first enteral feeding (*P *= 0.39). The GLP-1 curves of the groups following the first enteral feeding were statistically indifferent, as well (*P *= 0.60). Plasma fasting GLP-1 levels on the third day after the first enteral feeding did not differ between them (*P *= 0.91). The third day post-feeding GLP-1 curves of the groups were also statistically indifferent (*P *= 0.09) (Table 4).

**Table 3:** Documentation of median (minimum-maximum) GLP-1 measurements with regard to time points of enteral feeding both in Group 1 and Group 2

GLP-1 (pmol/L)	Group 1	Group 2	*P*
**Prefeeding**	5.3 (4.2 to 14.6)	7.5 (3.8 to 14.8)	0.384
**At 5 minutes after 1st feeding**	5.9 (4.1 to 12.0)	7.2 (4.3 to 12.4)	0.912
**At 15 minutes after 1st Feeding**	6.0 (3.5 to 17.2)	8.3 (4.0 to 12.9)	0.406
**At 30 minutes after 1st Feeding**	5.6 (3.9 to 10.6)	6.5 (3.7 to 13.3)	0.45
**At 60 minutes after the first feeding**	5.1 (4.4 to 10.6)	7.8 (4.9 to 12.5)	0.096
**At 120 minutes after the first feeding**	5.5 (4.3 to 10.8)	6.3 (4.3 to 16.1)	0.481
**Prefeeding on the third day**	7.3 (4.4 to 11.6)	6.9 (3.8 to 13.5)	0.912
**At 5 minutes after feeding on the third day**	7.6 (4.2 to 13.7)	6.4 (4.6 to 24.8)	0.971
**At 15 minutes after feeding on the third day**	8.3 (4.4 to 16.5)	6.6 (4.2 to 24.0)	0.971
**At 30 minutes after feeding on the third day**	8.5 (4.3 to 18.7)	7.2 (4.7 to 18.1)	0.912
**At 60 minutes after feeding on the third day**	6.6 (4.8 to 9.4)	9.2 (4.5 to 16.0)	0.143
**At 120 minutes after feeding on the third day**	5.9 (4.5 to 16.1)	6.2 (4.1 to 21.8)	0.091

**Table 4:** Mean GLP-1 levels in relation to enteral feeding in both groups

GLP-1 (pmol/L)	Group 1	Group 2	*P*
**Before first enteral Feeding**	7.10 ± 3.52	8.57 ± 3.91	0.38.9
*Following first enteral feeding	6.74 ± 2.39	7.68 ± 2.47	0.60
**On the third day before enteral feeding**	7.57 ± 2.84	7.84 ± 3.37	0.91
*On the third day following enteral feeding	7.85 ± 2.37	9.71 ± 4.46	0.09

*The measurements were performed at 5, 15, 30, 60 and 120 minutes of feeding

Plasma pre-feeding GLP-1 levels on the first and third day were compared with post-feeding GLP-1 curves for each group. There was no difference for Group 1 or for Group 2 between the pre-feeding GLP-1 levels and post-feeding GLP-1 curves on the first and third day (for Group 1; *P *= 0.97 and *P *= 0.10, for Group 2; *P *= 0.89 and *P *= 0.12, respectively).

The GLP-1 peak secretion time was 15 minutes after the first enteral feeding for both groups. According to the area under the curve (AUC) method, the GLP-1 peak level was 11.2 and 12.9 pmol/l for Group 1 and Group 2, respectively (*P *= 0.35).

**Figure 3:**
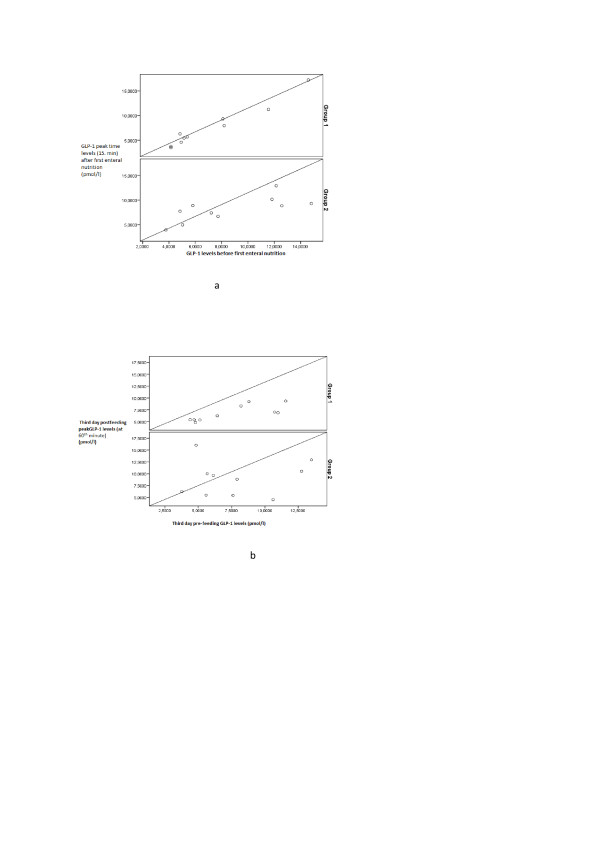
**a. First day pre-feeding GLP-1 and after feeding peak GLP-1 levels (at 15 minutes) in cases (*P *= 0.85 for Group 1, *P *= 0.98 for Group 2)**. **b**. Third day pre-feeding GLP-1 and after feeding peak GLP-1 levels (at 60 minutes) in cases (*P *= 0.63 for Group 1, *P *= 0.28 for Group 2).

The GLP-1 peak secretion time was 60 minutes for both groups on the third day after the first enteral feeding. Peak GLP-1 plasma levels exhibited statistically insignificant difference between Group 1 and Group 2; 18.7 vs 18.1 pmol/l, respectively (*P *= 0.49) (Figure 3).

### Changes in lymphocytes and lymphocyte subgroups

Baseline leucocyte, lymphocyte counts, T lymphocyte (CD3), T helper (CD3CD4), T cytotoxic (CD3CD8) and TREG cell amounts were similar between the two groups (*P *> 0.05). Twenty four hours after the first enteral feeding, T-helper and TREG cell amounts increased significantly in the early enteral feeding group (*P *= 0.02 and *P *= 0.036, respectively). However, similar increasing ratios were not observed in the late feeding group (*P *= 0.36 and *P *= 0.49 for T-helper and TREG cells, respectively). The number of T-cytotoxic cells decreased significantly in Group 1, but not in Group 2 (*P *= 0.0019 and *P *= 0.23, respectively) (Table 5).

**Table 5:** Comparison of hematologic parameters with exact number of lymphocytes and subgroups at *pre-feeding and **24 hours after first enteral feeding in Group 1 and Group 2

	Group 1 (*n *= 10)	*P*	Group 2 (*n *= 10)	*P*
**Leukocyte (cell/mm^3^)**	*12.02 ± 3.68 **13.00 ± 2.91	0.28	*10.74 ± 4.25 **11.14 ± 3.58	0.32
**Lymphocyte (cell/μl)**	*681.95 (321.9 to 3,745.4) **725.65 (130.9 to 4,620)	0.34	*688.2 (490.5 to 2,014) **845.4 (234 to 1,584)	0.18
**CD3 cells (cell/μl)**	*414.15 (167.39 to 2,857.74) **676.12 (82.1 to 2,684.22)	0.33	*476.8 (296.3 to 1,208.4) **549.7 (151.4 to 947.4)	0.65
**T helper cells (cell/μl)**	*229.17 (96.9 to 1,583.1) **469.22 (53.3 to 1,919.2)	0.02	*269.27 (136.4 to 821.7) **212.4 (61.4 to 636.6)	0.36
**T cytotoxic cells (cell/μl)**	*202.23 (24.6 to 746.7) **181.32 (55 to 1,123)	0.019	*168.99 (83.5 to 338.3) **171.4 (76.1 to 361.8)	0.23

**TREG cells (cell/μl)**	*0.22 (0 to 2.46) **0.41 (0.4 to 4.07)	0.036	*0.24 (0 to 1.14) **0.21 (0 to 0.53)	0.49

Data were given as the median (min-max) number of cells/3 mm or μl.

### Correlation between GLP-1 and lymphocyte changes

There was no correlation between the change in GLP-1 levels both on the first and the third day of enteral feeding, and the change in lymphocyte subgroups at baseline and 24 hours after feeding in Group 1 and Group 2 (Table 6).

**Table 6:** Documentation of the correlation analysis between GLP-1 change and change in lymphocyte subgroups (%) in all patients (*n *= 20)

GLP-1 (pmol/l) (*n *= 20)	T-helper cell change (%)	T-cytotoxic cell change (%)	TREG cell change (%)
**Baseline GLP-1**	*r *= - 0.19, *P *= 0.40	*r *= 0.28, *P *= 0.22	*r *= 0.035, *P *= 0.88
**GLP-1 curve following the first enteral feeding**	*r *= -0.45, *P *= 0.45	*r *= 0.43, *P *= 0.053	*r *= 0.21, *P *= 0.37
**GLP-1 curve following enteral feeding on the third day**	*r *= -0.47, *P *= 0.36	*r *= 0.45, *P *= 0.062	*r *= 0.17, *P *= 0.46

### Changes in plasma glucose levels

Glucose peak time obtained by the bedside glucometric method was the first measurement time (baseline fasting glucose) for both groups. The two groups exhibited statistically insignificant differences between the baseline glucose values and seven-day follow-up values obtained from AUC calculations (*P *= 0.47). There was negative correlation (50.1%) between the capillary glucose AUC and GLP-1 curve in all cases (*r *= -0.051, *P *= 0.039).

### Clinical implications

The NIH stroke scores exhibited statistically significant decline in both groups: *P *= 0.024 for Group 1 and *P *= 0.026 for Group 2. The amelioration in NIH scores began after the third day of hospitalization for all. The median percentage change of NIH stroke score in Group 1 was 6.7% and 6.5% for Group 2 (*P *= 0.26).

The median ICU stay of patients was 10 days (minimum: 3 to maximum: 29 days) in Group 1 and 15 days (minimum: 5 to maximum: 35 days) in Group 2, (*P *= 0.165).

One patient (10%) in Group 1 and four patients (40%) in Group 2 were diagnosed with infection (*P *= 0.30).

One patient in each group required mechanical ventilation (*P *= 0.10). After completion of study period one patient (10%) in Group 1 and two patients (20%) in Group 2 died (*P *= 0.50).

## Discussion

In this study, we did not detect any change in serial plasma GLP-1 measurements of ICU patients with acute thromboembolic stroke subjected to early and late enteral feeding performed via nasogastric route according to standard criteria [16]. There had also been no difference between the pre-feeding GLP-1 levels and enteral feeding GLP-1 curves of cases within the groups. We additionally demonstrated that early enteral feeding significantly increased the number of T helper and TREG cells, and decreased the amount of T cytotoxic cells with any change in plasma GLP-1.

We decided to perform this investigation on patients with acute thromboembolic stroke because of their acute illness status. Thereby, we aimed to avoid the impact of any interfering situation. Recently demonstrated neuroprotective properties of GLP-1 via GLP-1 receptors on neurons was the other reason [20-24].

Current study did not exhibit the presumed postprandial increase at GLP-1 levels in both early and late enteral feeding groups. These findings may be interpreted in two ways. The first explanation is the impact of meal kinetic effect on GLP-1 secretion which is determined principally by meal size [25]. Large amounts of nutrition have a greater impact on GLP-1 secretion than small amounts [26,27]. In our study, keeping in accordance with the ESPEN guideline for enteral nutrition [16,18], for avoiding the risk of aspiration pneumonia and feeding diarrhea, our patients were fed with standard amounts of a liquid mixed meal. Small amounts of the liquid meal, 10 to 30 cc at introduction, may not be sufficient for the stimulation of GLP-1 secretion. The second explanation is the possible physiological transient hypercortisolism originating from the stress caused by acute stroke. Studies on critically ill patients have demonstrated that low GLP-1 levels accompanying stress hyperglycemia is negatively correlated with plasma cortisol levels [28-30]. In a recent study, exogenous GLP-1 infusion has been demonstrated to attenuate stress hyperglycemia in ICU patients [31]. This study may underline the inadequacy of incretin response in ICU patients and support our findings.

Post-ischemic inflammation resulting from focal hypoperfusion-induced oxidative injury causes permanent neuronal damage in ischemic stroke [13]. Cellular immunity by T-lymphocytes plays a pivotal role in post-ischemic inflammation. T-regulatory cells (TREG) characterized by the expression of CD4CD25 surface marker and foxP3 transcription marker at flow-cytometry, are immune tolerance elements that inhibit the magnitude of immunopathological damage via decreasing the severity of T-cell mediated immune response and avoiding its permanency [32]. These cells have experimentally been shown to decrease infarct damage in acute stroke [33]. In recent animal models, the presence of GLP-1 receptors on T lymphocytes has been demonstrated and experimentally their stimulation has been shown to increase peripheral TREG cells [10,11]. Besides, factors other than GLP-1 that may act on TREG cells should not be ignored. The increasing number of TREG cells in our early enteral feeding group without accompanying GLP-1 elevation may be a good example of this proposal.

A limited number of studies investigating the influence of nutritional state and some micronutrients on cell-mediated immunity and early enteral nutrition have been shown to ameliorate cell-mediated immunity in ICU patients [34,35]. However, the impact of early enteral nutrition on TREG cells is obscure and there is no relevant study. In this trial, among ICU patients with acute stroke, we clearly demonstrated that early enteral nutrition resulted in augmentation of T helper and TREG cell amounts. T-helper cells are very well known to increase the TREG cell population via their secretory cytokines [32], so together the increase of these cell groups is not a surprise.

The clinical outcomes of early enteral feeding had also been investigated in the present study. Even though statistically insignificant, probably due to the low number of patients, median ICU stay and risk of infection tended to be lower with early enteral nutrition. This finding has been supported by a retrospective study performed on 4,049 critically ill, mechanically ventilated patients [36]. The decreasing number of cytotoxic T cells in our early enteral feeding group may be considered as a contradiction to the declining risk of infection in this group. Cytotoxic T cells are well-recognized members of cell-mediated immunity via their direct cytolytic effect [32]. It has been recently demonstrated that the immune modulatory effects of TREG cells are partially mediated by lowering the cytotoxic T cell number and thereby slowing the response rate of cell-mediated immunity [37,38]. Depending on the data mentioned above, we may propose that the decreasing cytotoxic T cell amounts in our early enteral feeding group may be related to their increasing TREG cell counts. In the same group, a decrease in risk of infection, in spite of declining T cytotoxic cells, may be attributed to the increase in T-helper cells, modulating humoral immunity via cytokines, and their impact on natural killer cells [32].

The link between early enteral nutrition and the increasing T-helper and TREG cell population was not via GLP-1 and blood glucose changes. The curves of blood glucose changes were similar between Group 1 and Group 2.

Early enteral nutrition has been shown to positively affect cellular immunity among surgical ICU patients [36]. In a study performed on neurological and non-neurological ICUpatients, GLP-1 levels have been found to be indifferent, as well [28]. According to these studies, we do not expect to see different results in various ICU patient groups except for cases with interfering factors.

There are a few pitfalls of the present study. The immune-modulating and neuroprotective properties of GLP-1 are reported primarily in animal models when pharmacological doses of GLP-1 (or an agonist) are administered. Hence, infusion of GLP-1 (or an agonist) would have been an alternative approach for our study. However, the decision of not infusing GLP-1 or a GLP-1 agonist into acutely-ill ICU patients was made, taking the ethical issues into consideration. As is known, GLP-1 infusion is not free of complications. For instance, it may increase the risk of aspiration pneumonia via decreasing gastrointestinal system (GIS) motility [31]. Deane *et al*. has shown that in critically ill patients with normal gastric emptying during placebo infusion, GLP-1 slowed gastric emptying substantially but, if the gastric emptying was delayed during placebo infusion, GLP-1 had no detectable effect on gastric emptying [39]. Therefore, the gastrointestinal effects of GLP-1 appear to be diminished in the critically ill.

Performing GLP-1 measurements at different time points, even though equally matched with regard to enteral feeding times, may be considered as a second limitation. This situation may be regarded as an inhomogeneity with regard to timing. Nevertheless, the statistically insignificant difference between the GLP-1 levels of both groups before the first enteral feeding, makes this inhomogeneity clinically worthless.

Parenteral nutrition is a potential and substantial confounder. It can be argued that the extra parenteral nutrition is the cause of the observed changes in lymphocyte subgroups and clinical implications, rather than early enteral nutrition in our study. In Casaer's study, the negative outcomes observed by introduction of parenteral nutrition early within the first two days when compared to late (after eight days) are attributed to the changes in blood glucose levels [40]. Accordingly, parenteral nutrition may be considered as an important factor when interpreting the results of such studies. In our study, taking the negative impact of malnutrition into account, the daily energy requirement was completed with enteral and parenteral nutrition where needed within each participant. The results might be confusing if there was a mismatch at the energy supply for both groups. In his study, Casaer defines late parenteral supply as "after 8 days". However, in our study, every participant received parenteral nutrition beginning on the first day. Mean glucose measurements of our groups were statistically indifferent both at inclusion and during the following seven days (*P *= 0.47). Finally, although the plasma samples were obtained at different time points of acute illness, mean GLP-1 levels of our groups before first enteral feeding were similar (*P *= 0.389). Lymphocyte counts and subgroups' amounts were also indifferent (shown in Table 5). Depending on the information mentioned above, it is reasonable to say that parenteral nutrition does not have a negative impact on our results.

## Conclusions

In our study the number of T-helper lymphocytes and TREG cells were found to increase with early enteral feeding in neurology ICU patients. The changes in lymphocyte subgroups were not related to GLP-1 levels. The clinically positive effects of lymphocyte changes were demonstrated, as well. Considering the lack of GLP-1 response of our patients to enteral feeding, we think that GLP-1-based therapies may have some additional benefits in this area of expertise. This hypothesis needs to be proven with further experimental and clinical studies.

## Key messages

• Serial plasma GLP-1 measurements exhibited statistically insignificant differences with early enteral feeding in acutely ill neurology ICU patients.

• The number of T helper and TREG cells were shown to increase and T cytotoxic cells were shown to decrease with early enteral feeding in acutely ill neurology ICU patients. However, the impact of early parenteral support given to complete the calculated daily energy requirement cannot be denied.

• Positive clinical effects of early enteral feeding on predisposition to infections and ICU stay time were observed.

• Cell-mediated immunity is assumed to ameliorate via factors other than GLP-1 among these patients.

## Abbreviations

AUC: Area under curve; ARDS: Adult respiratory distress syndrome; CD3: Total T lymphocytes; CD3CD4: T-helper cells; CD3CD8: Cytotoxic T cells; CD4+/CD25high/foxP3: TREG cells; CV: Coefficient of variation; DPP-IV: Dipeptidyl peptidase-IV; ESPEN: European Society of Parenteral and Enteral Nutrition; GIS: Gastrointestinal system; GLP-1: Glucagon-like peptide-1; ICU: Intensive care unit; NGT: Nasogastric tube; NIH: National Institute of Health; PN: Parenteral nutrition; ROC: Receiver operating characteristic; SIRS: Systemic inflammatory response syndrome; TREG: T regulatory.

## Competing interests

The authors declare that they have no competing interests.

## Authors' contributions

OB conceived and designed the study, and drafted the manuscript. EB helped to draft the manuscript, helped in acquisition and interpretation of data, and also edited the language. SG and ZA followed up on the patients in ICU. IK carried out the flow-cytometric analyses. NS carried out the GLP-1 assays. CS performed the statistical analysis. EE helped to draft the manuscript, helped in the design of the study, was involved in interpretation of data and in revising the manuscript for important intellectual content. All authors read and approved the final version of the manuscript.
